# A stochastic model of jaguar abundance in the Peruvian Amazon under climate variation scenarios

**DOI:** 10.1002/ece3.6740

**Published:** 2020-09-21

**Authors:** Kevin Burrage, Pamela Burrage, Jacqueline Davis, Tomasz Bednarz, June Kim, Julie Vercelloni, Erin E. Peterson, Kerrie Mengersen

**Affiliations:** ^1^ ARC Centre of Excellence for Mathematical and Statistical Frontiers, and Mathematical Sciences School Queensland University of Technology (QUT) Brisbane QLD Australia; ^2^ Department of Psychology University of Cambridge Cambridge UK; ^3^ Institute for Environmental Studies Vrije Universiteit Amsterdam Amsterdam The Netherlands

**Keywords:** climate change, jaguar, population, temporal model

## Abstract

The jaguar (Panthera onca) is the dominant predator in Central and South America, but is now considered near‐threatened. Estimating jaguar population size is difficult, due to uncertainty in the underlying dynamical processes as well as highly variable and sparse data. We develop a stochastic temporal model of jaguar abundance in the Peruvian Amazon, taking into account prey availability, under various climate change scenarios. The model is calibrated against existing data sets and an elicitation study in Pacaya Samiria. In order to account for uncertainty and variability, we construct a population of models over four key parameters, namely three scaling parameters for aquatic, small land, and large land animals and a hunting index. We then use this population of models to construct probabilistic evaluations of jaguar populations under various climate change scenarios characterized by increasingly severe flood and drought events and discuss the implications on jaguar numbers. Results imply that jaguar populations exhibit some robustness to extreme drought and flood, but that repeated exposure to these events over short periods can result in rapid decline. However, jaguar numbers could return to stability—albeit at lower numbers—if there are periods of benign climate patterns and other relevant factors are conducive.

## Graphical abstract

We develop novel statistical methods to address urgent conservation problems characterized by high levels of uncertainty. We demonstrate these methods by forecasting the effects of severe climate events on jaguar populations in the Peruvian Amazon basin.

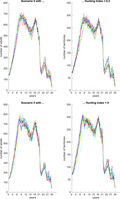

## 1 INTRODUCTION

The jaguar (Panthera onca) is the dominant predator in Central and South America and is important culturally and socially for many Indigenous American societies (Cristancho & Vining, 2004). Moreover, it has a positive impact on the environment since there is a high correlation between healthy carnivore populations and healthy habitats (Berger, Stacey, Bellis, & Johnson, 2001). However, the jaguar is now considered near‐threatened by the IUCN 2013 red list (Bernal‐Escobar, Payan, & Cordovez, 2015; Caso et al., 2011). It is now restricted to approximately 46% of its range in 1900 (Sanderson et al., 2002), with numbers being negatively impacted by a range of factors including loss and change of habitat, interaction with humans and declines in its prey base (Zeller, 2007).

Accurate estimation of the number and distribution of jaguars is difficult. They are cryptic by nature; they are not always uniquely identifiable and their habitat can be hostile to humans (Scognamillo, Maxit, Sunquist, & Polisar, 2003). Their range can vary considerably depending on gender, habitat, and prey availability, from 150km^2^ in the Cerrado region of Brazil (Silveira, 2004) to 36 km^2^ in Nicaragua (Zeller, Nijhawan, Salom‐Pore, Potosm, & Hines, 2011). Estimates can also be influenced by the sampling design, for example, the density of camera traps or size of the sampling units (Cuyckens, Falke, & Petracca, 2014; Sollmann et al., 2012), and by the method of analysis.

Given these challenges, computational modeling combined with sophisticated statistical techniques and a variety of data sources are becoming increasingly powerful mechanisms to evaluate jaguar population distributions (Sanderson et al., 2002). For example, Tobler et al. 2015 (Tobler, Hartley, Carrillo‐Percastegui, & Powell, 2015) used multiple camera trap surveys and multi‐session, multi‐species occupancy Royle‐Nichols models for three areas in Madre de Dios, Peru. Sollmann et al. (Sollmann et al., 2012) also used camera trap data and hierarchical models to show negative spatial correlations between Panthera onca (jaguars) and Felis concolor (pumas) in Emus National Park, Brazil. A different approach was adopted by Petracca (Petracca, 2010) based on interviews with local hunters and farmers in Toledo in South Belize to estimate both jaguars and prey presence. Interview data were also used by Zeller et al. (Zeller et al., 2011) who combined interview data with occupancy modeling for corridor identification in Nicaragua. This study also focused on estimates of abundances of seven main prey species for jaguar and used prior knowledge of the ecology and prey and jaguar behavior in building the models. This approach was also taken by Cuyckens et al. (Cuyckens et al., 2014) who studied possible corridors between Bolivia and Argentina through interviews regarding the presence of jaguars and six prey species. Examples of other modeling approaches include multi‐distance spatial clustering and logistic regression to estimate spatial abundance in Mato Grosso (Central Western Brazil) (Zeilhofer, Cezar, Torres, de Almeida Jocomo, & Silveira, 2014), and reaction–diffusion partial differential equations based on logistic growth to analyze jaguar sustainability under corridor development in Colombia (Bernal, Payon, & Cordovez, 2011) and a South America‐wide approach based on coupled reaction‐diffusion equation systems for male and female abundances.

Despite this body of literature, only a small fraction of published papers focus on prediction of jaguar numbers in the face of climate change, which is a major global concern for all threatened species (Thomas et al., 2004). An early example is Torres et al. (Torres, Marco, Diniz Filho, & Silveira, 2008) who used ecological niche modeling and the Mahalanobis distance method (Mahalanobis, 1936) to predict future jaguar distributions in Brazil. These authors noted that extinction risk from climate change is not clear for jaguars due to their adaptability to different prey. However, the simulations do suggest declines in abundance under climate change scenarios and that decreases in peripheral areas may lead to increased local extinction, which can be accentuated by deforestation. More recent studies include a paper by Cuyckens et al. (Cuyckens, Perovic, & Herron, 2017) who modeled the jaguar throughout its entire range using a maximum entropy (MaxEnt) approach and a report by Bodmer et al. (Bodmer et al., 2015) on the impacts of climate change on wildlife conservation in the Samiria river basin of the Pacaya Samiria National Reserve in Peru.

The overall aim of this paper is to use computational statistical and mathematical modeling calibrated by a range of information sources to explore the potential effect of climate variation on jaguar numbers. We focus our attention on the Pacaya Samiria National Reserve, an area of 20,800 km^2^ in the Loreto region of the Peruvian Amazon comprising mostly primary forest (Durand & McCaffrey, 1999). We introduce a stochastic temporal Markov model that allows for the evolution over time of seven variables representing the number of single individuals and females with young. These variables are influenced by external factors such as hunting and co‐evolving factors such as the availability of ten species of prey. The model is calibrated using three substantive information sources, including a published census study, relevant figures extracted from other published literature, and interview data from park rangers and other Indigenous inhabitants of the area. We then use this base model to make future predictions about jaguar numbers under six climate variation scenarios that mimic increasingly severe flood and drought events. These simulated predictions are obtained using a population of models approach that facilitates investigation of the underlying variability of the model estimates, quantification of uncertainty in the predictions, and probabilistic scenario evaluation.

The paper therefore has four research objectives. The first is to develop a stochastic temporal Markov model to describe the dynamics of jaguar abundance in Pacaya Samiria. The second is to demonstrate calibration of such a model using diverse, sparse information sources. The third is to employ a population of models approach to systematically investigate the effect of various future climate variation scenarios on jaguar numbers in this region. The fourth is to evaluate whether the approach presented here can provide a generic framework for modeling complex ecological problems using sparse information sources.

## 2 METHODS

### 2.1 Case study region

The Amazon is the largest contiguous block of habitat in the jaguar's range and is considered to be a stronghold of jaguar numbers (Zeller, 2007). Pacaya Samiria is a large National Reserve in the Peruvian Amazon, located southwest of Iquitos and close to the border with Brazil (Durand & McCaffrey, 1999). It is bordered by the Rio Maranon to the north and west and by the Rio Ucayali to the east and south, and is dominated by two large river systems, the Rio Pacaya and the Rio Samaria. Most of the Reserve is flooded primary forest with little deforestation apart from some pressures especially from oil and gas exploration on the southern border.

The wildlife and inhabitants of the river systems are subject to large seasonal fluctuations between the dry (July to November) and wet (December to June) seasons. In this flooding period, many of the land‐based animals migrate to higher level ground, known as restingas, and this makes hunting by predators in a typical wet season much easier than in the dry season; on the other hand, the herbivores have much greater competition between one another (Bodmer et al., 2014, 2015). Plants, wildlife, and humans have adapted to this regime so the system is stable, but climate variation is now starting to have significant impact on this stability (Bodmer et al., 2015). In the ten years from 2005 to 2014, there were two severe droughts in the dry seasons of 2005 and 2010, with 2010 being the driest on record (Espinoza et al., 2012), while 2009, 2011, and 2012 saw extreme flooding. In fact, 2009 was estimated as the largest flood on record, with 2011 exceeding 2009 and 2012 exceeding 2011. 2014 was a normal year. These floods can dramatically reduce the total area of the restingas with concomitant detrimental impact on herbivore numbers. On the other hand in a normal dry season, the depth of the Samiria is between 4 and 8 meters, but in 2010 it was only 1 meter deep (Bodmer et al., 2015). Consequently, aquatic abundancies can be severely affected in a dry season.

The Pacaya Samiria Reserve is managed by paid guards stationed fulltime throughout the park but mainly at specific Point of Vigilance (PV) stations along the two river systems (Durand & McCaffrey, 1999). Conservation management also includes engagement with Indigenous communities (Petracca, 2010). For example, the turtle sale certification scheme and restrictions on how much bush meat can be sold within Pacaya Samiria has given indigenous groups a stake in long‐term conservation management. However, there is evidence that climate variation has reduced both the bush meat market and fishing, putting more pressure on animal populations (Bodmer et al., 2015).

Putting these aspects in further context, the authors of (Bodmer et al., 2014) state that “results from long‐term research presented in this book clearly show that the Peruvian Amazon is undergoing dramatic impacts from climate change in lowland flooded forest ecosystems”. Furthermore, these results are consistent with the use of IPCC‐AR4 models on climate change in western Amazonia (Cook, Zheng, & Yoon, 2012). We note that the El Nino cycle arising in the Pacific and the Walker cycle arising in the southern Atlantic Ocean have different and subtle effects on different regions of the Amazon in terms of flood and drought periods.

### 2.2 Data sources

Three main data sources were used to develop and quantify the models. These comprised a published census study, general published information on jaguar ecology, and an elicitation study of Indigenous rangers in the Reserve.

The census study (Bodmer et al., 2014, 2015) was conducted in the period 2006–2014 using a variety of techniques including camera traps and scat analysis. The focus was on three regions along the Rio Samaria: the Cuenca Baja, centered around the ranger station PV Samiria and PV Shiringal, the Cuenca Media centered around PV2 Tacshacocha, and Cuenca Alta centered around PV3 Hungurahui. The first region includes the largest of the three Indigenous villages in the Park, San Martin, (near PV1 Samiria). The study included Inia geoffrensis (river dolphins), Caimaninae (caiman), fish, parrots, and both terrestrial and tree mammals. The animals fare very differently in response to the extreme flooding and the drought. All terrestrial ground dwelling animals (including Pecari tajacu (collared peccary) and Tayassu pecari (white‐lipped peccary), Mazama americana (red brocket deer) and Odocoileus virginianus (white‐tailed deer), Tapirus (tapir), Cuniculus (paca), Dasyprocta (agouti), Dasypodidae (armadillo), and Myrmecophaga tridactyla (giant anteater)) showed declines following consecutive years of extreme flooding. In the case of Tapirus, the decline was less; the authors suggested that one of the reasons for this is that they are rarely eaten by predators such as jaguars due to their size. For the remainder, the significant reduction in the total area of the restingas (up to a factor of four (Bodmer et al., 2015)) leads to fewer food resources, greater competition between species, and greater predator pressure as the terrestrial animals are less dispersed and easier prey. Arboreal wildlife are much less affected as they can escape the flooding, but the authors suggested that they may still be impacted by longer term effects of climate change due to changes in the plant community and plant availability. The impact on jaguars over this time of rapid change is less clear. Camera trap data suggested stable numbers in this period, but there were less data available compared with other animals. On the other hand, the authors note that when the Dasyprocta population declined substantively in 2014 this had a very significant negative impact on the Leopardus pardalis (ocelot) population. Furthermore, aquatic animals and birds all showed similar trends in response to drought. They were severely affected by the 2010 drought but numbers had recovered after two years of intensive flooding in 2011 and 2012. Bodmer et al. (Bodmer et al., 2015) estimated that approximately 2,000,000 mammals died as a result of extreme climate events in the Peruvian Amazon, which is two orders of magnitude greater than the impacts of human hunting.

Other data extracted from the census study reports (Bodmer et al., 2015; Espinoza et al., 2012) for modeling purposes include estimated total biomass of mammals in kg/km^2^ per annum over the period 2006 to 2012 (Table 1), estimates of individuals/km^2^ for selected prey for the period 2006 to 2014 plus the year 2000 (Table 2), and numbers of camera trap photographs per 1,000 camera days for selected prey (Table 3).

**Table 1:** Estimate of total biomass of mammals, in kg/km^2^

2006	2007	2008	2009	2010	2011	2012
440	400	740	520	380	320	150

**Table 2:** Estimated number of individuals per km^2^, for selected prey. “Deer” include Mazama americana (red brocket deer) and/or Odocoileus virginianus (white‐tailed deer). Common and local names for other prey are as follows: Tayassu pecari (white‐lipped peccary or huangana), Pecari tajacu (collared peccary or sajino), Mazama americana (red brocket deer), Odocoileus virginianus (white‐tailed deer), Tapirus (tapir), Dasyprocta (agouti). Some data were not available for 2012–2014

	2000	2006	2007	2008	2009	2010	2011	2012	2013	2014
Tayassu pecari	10.5	7	2.5	10	3.64	0.88	0.59	0.2	‐	‐
Pecari tajacu	2.4	0.09	0.42	0.38	0.19	0.05	0.01	0.01	‐	‐
Deer	0.5	0.13	0.21	0.08	0.21	0.12	0.04	0.01	‐	‐
Tapirus	0.001	0.012	0.009	0.013	0.08	0.003	0.001	‐	‐	‐
Dasyprocta	2.1	1.05	0.6	0.65	1.2	1.07	0.9	0.39	0.1	0.015

**Table 3:** Number of camera trap photographs per 1,000 camera days, for selected animals. Common or local names for listed animals are as follows: Cuniculus (paca), Myrmecophaga tridactyla (anteater), Dasypodidae (armadillo), Tapirus (tapir), Leopardus pardalis (ocelot), Panthera onca (jaguar), Felis concolor (puma). Some data were not available for 2009

	2009	2011	2013	2014
Cuniculus	113	45.6	21.8	7.3
Myrmecophaga tridactyla	22.7	8.7	4.95	2.4
Dasypodidae	127.27	31.34	4.95	6.09
Tapirus	22.73	19.15	36.63	30.45
Leopardus pardalis	9.09	54.55	19.8	8.52
Panthera onca	‐	12.18	4.9	10.96
Felis concolor	‐	12.18	14.85	4.87

Bodmer et al. (Bodmer et al., 2014, 2015) draw a number of conclusions from their data. The first is that aquatic wildlife, in general, was negatively impacted by the 2010 drought, while consecutive years of flooding saw returns to healthy population levels. Secondly, terrestrial ungulates, rodents, and edentates including Tayassu pecari and Pecari tajacu, Mazama americana, Cuniculus, Dasyprocta, Myrmecophaga tridactyla, and Dasypodidae were negatively impacted by flooding as dry ground has been reduced. This was due to a reduction in primary food sources of fruits and seed and greater predation from carnivores. Thirdly, Tapirus numbers seem to have been less impacted perhaps because of its diversified diet. Fourthly, based on the camera trap data in Table 3, there is considerable variability in both the predator and prey populations, with some evidence of predator–prey relationships; for example, numbers of Felis concolor reduced substantially in 2014 after a dramatic reduction in their main prey, Dasyprocta.

The second source of information used to calibrate the model included a range of published data about jaguars. Regarding biology, we adopted the following figures: the average lifespan of the jaguar is 12 to 15 years (Bernal‐Escobar et al., 2015); the period of sexual maturity lasts 12.5 years (Bernal‐Escobar et al., 2015); the inter birth arrival is 2 years (Carrillo, Saenz, & Fuller, 2009); the male to female birth ratio is 1:2 (Tobler et al., 2015); and the typical number of offspring is 1 or 2 (Crawshaw & Quigley, 2002). Regarding weight, estimates vary from around 31 kg for females and 37 kg for males (Emmons, 1987) to 57–113 kg for males and 45–90 kg for females (Denver Zoo; livescience.com, Sept 22, 2017). Based on these ranges, and using an estimated prey consumption of 34–43 grams of mammalian meat per day per kg (Emmons, 1987), two sets of values were adopted for the daily meat consumption for an adult male, adult female, and juvenile: a lower bound of 1.4, 1.15 and 0.5 kg/day respectively, and an upper bound of 5, 4 and 2 kg/day, respectively. Regarding behavior, we assumed that the male territory is twice that of the female and there is general overlap of one male to two females (Crawshaw & Quigley, 1991). Reported density estimates included 2.67 ± 1 jaguars per 100 km^2^ in the semi‐arid Caatinga biome of north‐eastern Brazil (Silveira et al., 2010; Silver et al., 2004), 4.4 ± 0.7 jaguars per 100km^2^ in the department of Madre de Dios (Tobler et al., 2015), and 6 jaguars per 100 km^2^ in the Pantanal (Soisalo & Cavalcanti, 2006). For the number of individuals required for a genetically stable population, Sanderson et al (Sanderson et al., 2002) suggest that 50 individuals in a suitable habitat of approximately 1,000 km^2^ are not unreasonable.

The third source of data was based on an interview study conducted by the authors during a three week field trip in Pacaya Samiria via the Rio Samiria from Nauta, as far as the Rio Santo Elena, a tributary of the Rio Samaria just past PV5 St Elena. The route was recorded through an inReach Explorer satellite phone with built‐in navigation with waypoints and routing (inREACH Explorer, 2020). We recorded interviews with fifteen park rangers at the PV stations, six groups of Indigenous travelers who had stopped at the PV stations, and the six Indigenous members of the expedition team. The interviews were based on a small set of structured questions about jaguar encounters, including who experienced the encounter (gender, age, occupation, residence, experience in the forest), the location of the encounter (verbal description and recorded by a star on a map), the date and time of the encounter and the nature of the encounter (sight, sound, scat, etc.). This was followed by a set of structured questions about perceived trends in jaguar numbers over time and factors that affect jaguar numbers. The interview concluded with an unstructured conversation about jaguar‐related experiences. Overall, the data were relatively uniform about each of the sites, but there were slightly more reported encounters in the deeper areas of the reserve, where the jungle is more dense.

In the field expedition, we did not reach PV6 Hamburgo, which is very deep into Pacaya Samiria, but one of our guides, Robinson Huanucari, had been born and raised in that area and was the head ranger at this station at the time of our study. Based on his observations, he estimated that there were six permanent jaguar groups in the region of PV Hamburgo, which he patrolled in a dugout canoe with an outboard motor, up to a distance of about 5 hr from the station. Given the speed of the canoe and the tortuosity of the river systems, we estimate this area as approximately 400 km^2^. This would equate to a single territory size of approximately 70 km^2^ per group. Other parts of Pacaya Samiria are on higher ground and away from the main rivers. There were no guards stationed in these areas, and there is very little information available and considerable doubt as to whether the same territory size is a reasonable estimate in these regions. On the other hand, much of this land is higher and becomes restingas in the rainy season, which would naturally attract animals and hence jaguars. Given a park size of 20,000 km^2^, we adopted an estimate of about 300 territories with one male and two females per territory (Crawshaw & Quigley, 1991). Finally, the interviews revealed that due to major shifts in the management of Pacaya Samiria in the last decade, Indigenous groups have increasingly been given specific areas to manage in an attempt to reduce both internal and external poaching. There has been some success in this regard but it is clear that increased climate variation can only exacerbate these human pressures and their impact on predator and prey numbers. As discussed later, we attempt to capture the uncertainties due to human pressures through an explicit parameter in our model.

### 2.3 Building the model

We built a dynamic temporal stochastic model of jaguar numbers in Pacaya Samiria. The model, which was developed in Matlab, takes into account solitary behavior, mating, births of cubs at certain times of the year, competition from other animals including jaguars themselves for territories with concomitant reduction in birth rate, illegal hunting by humans and death from starvation. In the latter context, we took into account the hunting behavior of jaguars of 10 key prey at different times of the year, as described below.

The model was developed under the assumption that Pacaya Samiria is well‐managed and well‐protected with mostly pristine jungle habitat in which there is little hunting of jaguar within the reserve. Hence, we assumed that there is very little spatial variation in jaguar population across the reserve. Consequently, a stochastic temporal modeling framework was considered to be appropriate.

The model has 7 variables that evolve through time, namely single male (denoted by $S_{M}$, female without cub ($F_{0}$), female with 1 male cub ($F_{M}$), female with 1 female cub ($F_{F}$), female with 2 male cubs ($F_{\mathrm{MM}}$), female with a male cub and a female cub ($F_{\mathrm{MF}}$), and female with 2 female cubs ($F_{\mathrm{FF}}$). These variables are represented in a vector $S\left(t\right)$ as

$$S={\left(S_{M},F_{0},F_{M},F_{F},F_{\mathrm{MM}},F_{\mathrm{MF}},F_{\mathrm{FF}}\right)}^{T}.$$

The specific 28 transitions among these variables are described below in terms of four main events, namely birth of one or two cubs, death of an adult, death of one or two cubs, and departure of a cub from its mother as follows. It is assumed that the death of a female with cubs implies the death of the cubs as well.

**Table:** 

Birth	Adult Death	Cub Death	Split
$F_{0}\to F_{M}$	$S_{M}\to 0$	$F_{M}\to F_{0}$	$F_{M}\to F_{0}+S_{M}$
$F_{0}\to F_{F}$	$F_{0}\to 0$	$F_{F}\to F_{0}$	$F_{F}\to F_{0}+F_{0}$
$F_{M}\to F_{\mathrm{MM}}$	$F_{M}\to 0$	$F_{\mathrm{MM}}\to F_{M}$	$F_{\mathrm{MM}}\to F_{M}+S_{M}$
$F_{M}\to F_{\mathrm{MF}}$	$F_{F}\to 0$	$F_{\mathrm{MF}}\to F_{M}$	$F_{\mathrm{MF}}\to F_{M}+F_{0}$
$F_{F}\to F_{\mathrm{MF}}$	$F_{\mathrm{MM}}\to 0$	$F_{\mathrm{MF}}\to F_{F}$	$F_{\mathrm{MF}}\to F_{F}+S_{M}$
$F_{F}\to F_{\mathrm{FF}}$	$F_{\mathrm{MF}}\to 0$	$F_{\mathrm{FF}}\to F_{F}$	$F_{\mathrm{FF}}\to F_{F}+F_{0}$
	$F_{\mathrm{FF}}\to 0$		
		$F_{\mathrm{MM}}\to F_{0}$	
		$F_{\mathrm{MF}}\to F_{0}$	
		$F_{\mathrm{FF}}\to F_{0}$	

The 7×28 update (transition) matrix, denoted by v, is therefore

$$\left[\begin{array}{cccccccccccccc} 0 & 0 & 0 & 0 & 0 & 0 & ‐1 & 0 & 0 & 0 & 0 & 0 & 0 & 0\\ ‐1 & ‐1 & 0 & 0 & 0 & 0 & 0 & ‐1 & 0 & 0 & 0 & 0 & 0 & 1\\ 1 & 0 & ‐1 & ‐1 & 0 & 0 & 0 & 0 & ‐1 & 0 & 0 & 0 & 0 & ‐1\\ 0 & 1 & 0 & 0 & ‐1 & ‐1 & 0 & 0 & 0 & ‐1 & 0 & 0 & 0 & 0\\ 0 & 0 & 1 & 0 & 0 & 0 & 0 & 0 & 0 & 0 & ‐1 & 0 & 0 & 0\\ 0 & 0 & 0 & 1 & 1 & 0 & 0 & 0 & 0 & 0 & 0 & ‐1 & 0 & 0\\ 0 & 0 & 0 & 0 & 0 & 1 & 0 & 0 & 0 & 0 & 0 & 0 & ‐1 & 0 \end{array}\right)$$

$$\left(\begin{array}{cccccccccccccc} 0 & 0 & 0 & 0 & 0 & 0 & 0 & 0 & 1 & 0 & 1 & 0 & 1 & 0\\ 1 & 0 & 0 & 0 & 0 & 1 & 1 & 1 & 1 & 2 & 0 & 1 & 0 & 1\\ 0 & 1 & 1 & 0 & 0 & 0 & 0 & 0 & ‐1 & 0 & 1 & 1 & 0 & 0\\ ‐1 & 0 & 0 & 1 & 1 & 0 & 0 & 0 & 0 & ‐1 & 0 & 0 & 1 & 1\\ 0 & ‐1 & 0 & 0 & 0 & ‐1 & 0 & 0 & 0 & 0 & ‐1 & 0 & 0 & 0\\ 0 & 0 & ‐1 & ‐1 & 0 & 0 & ‐1 & 0 & 0 & 0 & 0 & ‐1 & ‐1 & 0\\ 0 & 0 & 0 & 0 & ‐1 & 0 & 0 & ‐1 & 0 & 0 & 0 & 0 & 0 & ‐1 \end{array}\right].$$

The probability of each of these four events (birth, adult death, cub death, split) is assumed to occur with probability ($k_{1},k_{2},k_{3},k_{4}$), respectively.

This dynamic temporal stochastic representation is a discrete‐time Markov model, meaning that the outcome of the system $S\left(t\right)$ at each time epoch *t* is only dependent on the state of the system at the previous epoch t‐1 and not on previous epochs. There are a number of ways of simulating the evolution of such a Markov model. One of the most common approaches is based on the Stochastic Simulation Algorithm (Gillespie, 1976) in which at time point *t* an exponential waiting time $\tau_{t}$ to the next transition is simulated, then the most likely transition, *j*, in that time window is chosen and the state vector is updated as $S\left(t+\tau_{t}\right)=S\left(t\right)+\nu_{j}$. However, this is not suitable for this model as it requires an exponentially distributed waiting time and this would imply too fine a time scale. Instead, we use the tau leap algorithm (Gillespie, 2001). In this algorithm, all the (*m*) transitions are allowed to happen in a given timestep τ, with a frequency based on the Poisson distribution, that can be chosen to be fixed, as long as there are not too many transitions in that step. The update formula is given by

(1) $$S_{n+\tau }=S_{n}+\sum_{j=1}^{m}\nu_{j}P\left(a_{j}\left(S_{n}\right)\tau \right)$$

where $P\left(\lambda \right)$ denotes the Poisson distribution with intensity λ. Here, $S_{n}$ denotes the value of S at $t=t_{n}$ and the propensity functions, $a_{j}\left(S\left(t\right)\right),j=1,\cdots ,28$, which describe the relative probability of each interaction occurring, are given as a vector:

$$\begin{array}{c} (k_{1}F_{0},k_{1}F_{0},k_{1}F_{M},k_{1}F_{M},k_{1}F_{F},\\ k_{1}F_{F},k_{2}S_{M},k_{2}F_{0},k_{2}F_{M},k_{2}F_{F},\\ k_{2}F_{\mathrm{MM}},k_{2}F_{\mathrm{MF}},k_{2}F_{\mathrm{FF}},k_{3}F_{M},\\ k_{3}F_{F},k_{3}F_{\mathrm{MM}},k_{3}F_{\mathrm{MF}},k_{3}F_{\mathrm{MF}},\\ k_{3}F_{\mathrm{FF}},k_{3}F_{\mathrm{MM}},k_{3}F_{\mathrm{MF}},k_{3}F_{\mathrm{FF}},\\ k_{4}F_{M},k_{4}F_{F},k_{4}F_{\mathrm{MM}},k_{4}F_{\mathrm{MF}},k_{4}F_{\mathrm{MF}},k_{4}F_{\mathrm{FF}}) \end{array}$$

We chose the time units to be in months and so take τ=1 month, as being a reasonable time step in which to update population counts.

2.4

1. It is sometimes possible for some elements of $S_{n+\tau }$ to become negative when some of the components of *S* are small and the step size is too large. We remedy this by setting

$$a_{j}\left(S_{n}\right)=0,a_{j}\left(S_{n}\right)<0$$

and

$$S_{n+\tau }^{j}=0,s_{n+\tau }^{j}<0,$$

for any *j* for which this occurs.

2. The average lifespan of the jaguar is between 12 and 15 years, and we will assume that most attain that age under good conditions. In order to reflect the fact that the age distribution of jaguars is approximately normal and that there is a much greater chance of older jaguars dying, we assume the death rate is an appropriately scaled linear combination (using two different uniform random numbers) of $\frac{0.975}{\mathrm{nd}}$ and $\frac{0.025}{12}$, where *nd* is the average lifespan of a jaguar times the period of the simulation.

3. Given our estimate of the number of jaguar territories as 300 and assuming that the birth rate is constrained by the number of territories, we assume that the birth rate depends on time and takes the form

$$k_{1}\left(t\right)=\frac{1}{4}\left(1‐{\left(\frac{NumT\left(t\right)}{300}\right)}^{P}\right).$$

With P=4, the birth rate is relatively flat over a large range of the number of occupied territories, $NumT\left(t\right)$, so $k_{1}\left(t\right)$ only approaches zero as the threshold of the maximum number of territories is approached. We also assume that births only occur in the four months: July, August, September, October, before the start of the rainy season, hence the scaling factor in $k_{1}\left(t\right)$.

We set the cub release rate as $k_{0}=\frac{1}{18}$, to indicate that after $1\frac{1}{2}$ years the cub leaves the female.

5. Since we were unable to find satisfactory information about hunting of jaguars by humans in the case study region, we introduce a hunting index $H\in \left[0,1\right]$ such that that the integer part of *H* times the jaguar population is removed from the population at the end of the year. The value of *H* is set external to the model. Two values of *H* are considered here, namely H=0 (representing no hunting pressure) and H=0.2 (indicating that 1 in 5 available jaguars are killed by humans).

One of the features of our model is a detailed representation of the prey of the jaguar. We identified 10 animal sources:(1) fish (e.g., arapaima (paiche)); (2) turtles (e.g., Podocnemis expansa (Amazon River turtle) and Podocnemis unifilis (yellow‐spotted river turtle); (3) Dasypodidae (armadillo); (4) Dasyprocta (agouti); (5) Hydrochoerus hydrochaeris (capybara); (6) Cuniculus (paca); (7) Tapirus (tapir); (8) Pecari tajacu (collared peccary or sajino); (9) Tayassu pecari (white‐lipped peccary or huangana); (10) deer (e.g., Mazama americana (red brocket deer) and Odocoileus virginianus (white‐tailed deer). Based on information provided by Indigenous residents of the area, the land‐based animals are mainly attracted by fruiting trees and jaguars patrol these areas at appropriate times of the year. We constructed a matrix with 10 rows and 12 columns (the months) when these animals are available. The matrix is

$$\begin{array}{ccccccccccccc} & 1 & 2 & 3 & 4 & 5 & 6 & 7 & 8 & 9 & 10 & 11 & 12\\ 1 & \theta_{1} & \theta_{1} & \theta_{1} & \theta_{1} & \theta_{1} & \theta_{1} & \theta_{1} & \theta_{1} & \theta_{1} & \theta_{1} & \theta_{1} & \theta_{1}\\ 2 & 0 & 0 & 0 & 0 & 0 & \theta_{2} & \theta_{2} & \theta_{2} & \theta_{2} & \theta_{2} & 0 & 0\\ 3 & \theta_{3} & \theta_{3} & \theta_{3} & \theta_{3} & \theta_{3} & \theta_{3} & \theta_{3} & \theta_{3} & \theta_{3} & \theta_{3} & \theta_{3} & \theta_{3}\\ 4 & \theta_{4} & \theta_{4} & \theta_{4} & \theta_{4} & \theta_{4} & 0 & 0 & 0 & 0 & 0 & 0 & \theta_{4}\\ 5 & \theta_{5} & \theta_{5} & \theta_{5} & \theta_{5} & \theta_{5} & \theta_{5} & \theta_{5} & \theta_{5} & \theta_{5} & \theta_{5} & \theta_{5} & \theta_{5}\\ 6 & \theta_{6} & \theta_{6} & \theta_{6} & \theta_{6} & \theta_{6} & 0 & 0 & 0 & 0 & 0 & 0 & \theta_{6}\\ 7 & \theta_{7} & \theta_{7} & \theta_{7} & \theta_{7} & 0 & 0 & 0 & 0 & 0 & 0 & 0 & 0\\ 8 & \theta_{8} & \theta_{8} & \theta_{8} & \theta_{8} & 0 & 0 & 0 & 0 & 0 & 0 & 0 & \theta_{8}\\ 9 & \theta_{9} & \theta_{9} & \theta_{9} & \theta_{9} & 0 & 0 & 0 & 0 & 0 & 0 & 0 & \theta_{9}\\ 10 & 0 & \theta_{10} & \theta_{10} & \theta_{10} & 0 & 0 & 0 & 0 & 0 & 0 & 0 & 0. \end{array}$$

We estimated these θ based on relative abundances, frequency of hunting, and average weight of the animal. We used the following average weight in kgs: fish (1), (40), turtles (10, based on 40 for Podocnemis expansa and 4 for Podocnemis unifilis), Dasypodidae (4), Dasyprocta (4), Hydrochoerus hydrochaeris (40), Cuniculus (10), Tapirus (150), Pecari tajacu (10), Tayassu pecari (20) and Mazama americana and Odocoileus virginianus (30). These figures were based partly personal communication from Indigenous residents of the area and partly extracted from (Bodmer et al., 2014, 2015).

In order to make further simplifications, we classified the θ into just three categories: $\theta_{\mathrm{aquatic}}$ (consisting of fish and turtles), $\theta_{land\_small}$ (Dasypodidae and Dasyprocta), and $\theta_{land\_large}$ (Hyrdochoerus hydrochaeris, Cuniculus, Tapirus, Pecari tajacu, Tayassu pecari, Mazama americana, Odocoileus virginianus). The reason for this is that extreme flooding or droughts will affect the aquatic and land‐based animals very differently, and in addition, the small land‐based and large land‐based animals will also fare differently (Bodmer et al., 2014).

Based on this, we introduced a hunger index due to lack of prey, based on the prey table. For a given month, we summed the $\theta_{i}$ and compared that sum with an estimate of the total food that the population of jaguars would consume in that month given previously. Hence, we defined a food ratio R. If R<1, then there is enough food for that month. A value of R>1 affects the death rate by multiplying by R the natural death rate. If the product of these rates over two consecutive months is greater than some specified threshold (30), then this counts as a catastrophic event. The male jaguar kills any cubs and the birth rate is set to 0.

### 2.5 Model parameters

Our model has 4 parameters over which we explored the dynamics. These are the hunting index H and parameters $K_{A}$, $K_{\mathrm{SL}}$, $K_{\mathrm{LL}}$. These three latter parameters relate the fraction of the total mass of aquatic, small land, and large land animals available to the jaguars.

Two values of H are considered (see Remark (v) above). In order to account for the intrinsic variability in abundance numbers as a function of these parameters and the lack of detailed information about the effects of human pressure in the reserve, we evaluate multiple values of $K_{A}$, $K_{\mathrm{SL}}$, and $K_{\mathrm{LL}}$ based on a population of models approach (Britton et al., 2013; Burrage, Burrage, Donovan, & Thompson, 2015; Marder & Taylor, 2011). Under this approach, values of the three parameters are obtained via Latin Hypercube sampling (McKay, Beckman, & Conover, 1979) over appropriate ranges of the parameters. Latin Hypercube sampling is a grid‐based approach that provides a near‐random sample of the parameter space. It provides good coverage of parameters and does not scale with dimension (Burrage et al., 2015). Given the 4 parameter values, we run a relatively small number of simulations of the stochastic model and we accept the model (and hence the set of parameters) if all the simulations are stable in some appropriate range and are consistent with the data. Using this approach, we can characterize the distribution of parameters associated with various populations of models under various climate change scenarios.

### 2.6 Climate variation scenarios

Initially, we calibrated the model against so‐called “normal” years over the period 2000–2008. We assumed that there is adequate prey available so that both prey and jaguar numbers are stable. Consequently, we also assumed the hunting index H was zero for this initial calibration. Although we did not have abundance figures for all animals, we used the data in Tables 1 and 2 scaled by the typical animal masses for small, large, and aquatic animals. We then multiplied these by a typical territory size or river length within that territory. This led to relative values of $\theta_{\mathrm{aquatic}}=1$, $\theta_{land\_small}=2$, and $\theta_{land\_large}=4.5$ for aquatic, small land animals, and large land animals, respectively. We also defined scaling factors, $S_{A}$, $S_{S}$, and $S_{L}$, for each category, respectively, based on the relative biomass of the jaguar prey. For the period 2000 – 2008, these scale factors, $S_{A}$, $S_{S}$, and $S_{L}$, were set to 1. We then considered the period 2009 – 2014. Based on evidence of the camera traps, we assumed that even under this regime jaguar numbers are still stable, but they may not be at their maximum carrying capacity. Based on the previous tables and information in (Bodmer et al., 2014, 2015) on aquatic numbers after droughts, we took the values for the scale factors as shown in Table 4.

**Table 4:** Scaling factors $S_{A}$, $S_{S}$ and $S_{L}$ for aquatic, small, land and large land animals, respectively, over 6 years encompassing one drought (2010) and three floods (2009, 2011, 2012)

	2009	2010	2011	2012	2013	2014
$S_{A}$	1	$\frac{1}{5}$	$\frac{1}{2}$	1	1	1
$S_{S}$	$\frac{52}{74}$	$\frac{38}{74}$	$\frac{32}{74}$	$\frac{15}{74}$	$\frac{10}{74}$	$\frac{8}{74}$
$S_{L}$	$\frac{52}{74}$	$\frac{38}{74}$	$\frac{32}{74}$	$\frac{15}{74}$	$\frac{10}{74}$	$\frac{8}{74}$

Here we have assumed that climate variability affects small and large land‐based animals in the same way, but aquatic animals are affected only by drought (and not floods) and return to normal values within two years of an extreme drought (Bodmer et al., 2015).

In the simulations, we made the following assumptions. The drought affects aquatic numbers only over a two year period with scale factors (1/5,1/2), see Table 4, but then the scale factors will return to the previous state. On the other hand, if another flood occurs before normality is restored then the last scale factor will be multiplied by this pair for the following two years. There is no effect on aquatic numbers through floods. In the case of small and large territorial animals, we assume that both are equally affected by flood and drought. In the case of a flood, it is difficult to separate out a single event from the three floods in 2009, 2011, and 2012, but a reasonable approximation based on Table 4 is that a single flood has scale factors (1/3,1/2,1/3) over three years and then return to the previous state. In the case of drought, we assume that the scale factor in the following year is 0.7 (approximately 5/7) and then returns to 1 for both large and small animals.

We commenced the study by calibrating the model over the 15 years (2000 to 2014) with hunting index H=0 and first all the scale factors as 1 (Scenario 1), and secondly with the animal scaling factors as given in Table 4 for the years 2009 to 2014, characterized by one drought and three floods (Scenario 2). We repeated this with a hunting index of H=0.2. In both cases, the three animal scaling factors were set to 1. This provides a baseline predicated on the historical data. The initial conditions of jaguar numbers were set relatively low to show that the numbers can quickly reach the maximum carrying capacity under benign conditions.

We then followed on directly from Scenario 2 and considered a projection 15 years into the future under 4 scenarios. As above, two hunting indices of 0 and 0.2 were considered. The effects of drought and floods are based on the data from Table 3. The 4 scenarios are described below, with tables showing the scaling factors for $S_{A}$ and $S_{L}$ ($S_{S}=S_{L}$) for each of the 15 future years.

**Scenario 3:** In this scenario, the scale factors for $S_{S}$ and $S_{L}$ gradually return to 1 over a 4‐year period as the effects of the floods and droughts from 2009 to 2014 are mitigated. This is then followed by five years of stable behavior and then an alternating sequence of extreme floods and droughts every second year, characterized as:

**Table:** 

$S_{A}$	1	1	1	1	1	1	1	1	1	$\frac{1}{5}$	$\frac{1}{2}$	$\frac{1}{10}$	$\frac{1}{4}$	$\frac{1}{20}$	$\frac{1}{8}$
$S_{S}=S_{L}$	$\frac{10}{74}$	$\frac{15}{74}$	$\frac{32}{74}$	$\frac{52}{74}$	1	1	1	1	1	$\frac{32}{74}$	$\frac{15}{74}$	$\frac{7}{74}$	$\frac{3}{74}$	$\frac{1}{74}$	$\frac{1}{148}$

**Scenario 4:** In this scenario, there is initially a sequence of droughts over 6 years then a flood and then two successive droughts, characterized as:

**Table:** 

$S_{A}$	$\frac{1}{5}$	$\frac{1}{2}$	$\frac{1}{10}$	$\frac{1}{4}$	$\frac{1}{20}$	$\frac{1}{8}$	$\frac{1}{4}$	1	1	1	1	$\frac{1}{5}$	$\frac{1}{2}$	$\frac{1}{10}$	$\frac{1}{4}$
$S_{S}=S_{L}$	$\frac{15}{74}$	$\frac{32}{74}$	$\frac{52}{74}$	1	1	1	1	$\frac{32}{74}$	$\frac{15}{74}$	$\frac{10}{74}$	$\frac{32}{74}$	1	1	1	1

**Scenario 5:** In this scenario, there is an alternating sequence of drought and flood with stable climate behavior in between these extreme events so that prey numbers may partially recover, but never fully, characterized as

**Table:** 

$S_{A}$	$\frac{1}{5}$	$\frac{1}{2}$	$\frac{1}{10}$	$\frac{1}{4}$	$\frac{1}{5}$	$\frac{1}{2}$	$\frac{1}{10}$	$\frac{1}{4}$	$\frac{1}{20}$	$\frac{1}{8}$	$\frac{1}{10}$	$\frac{1}{4}$	$\frac{1}{20}$	$\frac{1}{8}$	$\frac{1}{40}$
$S_{S}=S_{L}$	$\frac{15}{74}$	$\frac{32}{74}$	$\frac{52}{74}$	$\frac{32}{74}$	$\frac{15}{74}$	$\frac{7}{74}$	$\frac{3}{74}$	$\frac{7}{74}$	$\frac{15}{74}$	$\frac{7}{74}$	$\frac{3}{74}$	$\frac{1}{74}$	$\frac{3}{74}$	$\frac{1}{74}$	$\frac{1}{148}$

**Scenario 6:** In this scenario, there is initially an alternating sequence of drought and flood where prey numbers may partially recover but never fully. In the final 5 years, there are successive years of drought and flood with no chance of recovery. This is characterized as

**Table:** 

$S_{A}$	$\frac{1}{5}$	$\frac{1}{2}$	$\frac{1}{10}$	$\frac{1}{4}$	$\frac{1}{5}$	$\frac{1}{2}$	$\frac{1}{10}$	$\frac{1}{4}$	$\frac{1}{20}$	$\frac{1}{8}$	$\frac{1}{40}$	$\frac{1}{16}$	$\frac{1}{80}$	$\frac{1}{32}$	$\frac{1}{160}$
$S_{S}=S_{L}$	$\frac{15}{74}$	$\frac{32}{74}$	$\frac{52}{74}$	$\frac{32}{74}$	$\frac{15}{74}$	$\frac{7}{74}$	$\frac{3}{74}$	$\frac{7}{74}$	$\frac{15}{74}$	$\frac{7}{74}$	$\frac{3}{74}$	$\frac{1}{74}$	$\frac{3}{148}$	$\frac{1}{148}$	$\frac{1}{296}$

We compared the scenarios by building a population of models for Scenarios 2, 3, and 5. Since the hunting index was shown to have a relatively small influence when jaguar numbers are small (see Results section), we built populations of models on the three scaling parameters that lie in the space ${\left[0,1\right]}^{3}$. We sampled this space using Latin hypercube sampling with n=50, so that the grid resolution is 0.02. For each chosen cell, we sampled uniformly at random and we perform five different trials. A model was placed in the population if for a given parameter choice all simulations were above the prescribed percentage of the minimum carrying capacity.

### 2.7 Sensitivity assessment

A sensitivity assessment was undertaken to evaluate the effect on the two outcomes of interest (number of animals, number of regions) of key assumptions in the above scenarios. These assumptions related to the following characteristics of the jaguar: the size of the home range (and hence the number of available territories), the average age of the animal, the duration of the reproductive season, the hunger threshold, and the food requirements. Five experiments were conducted, in which each characteristic was varied in two ways, keeping other variables at the assumed values. The experiments are described in Table 5.

**Table 5:** Sensitivity assessment experiments to test model assumptions. Vector *F* is kg/day for adult male, adult female, cub

Expt. No.	Characteristic	Assumed value	Change
1	Number of territories	300	Half, Double
2	Average age of animal	10 years	15 years
3	Reproductive season	3 months	Double
4	Hunger threshold	Nominal 30	Half, Double
5	Food requirement	*F* = [5, 4, 2]	Half, Double

width = 0.90

## 3 RESULTS

### 3.1 Climate variation scenarios

**Figure 1:**
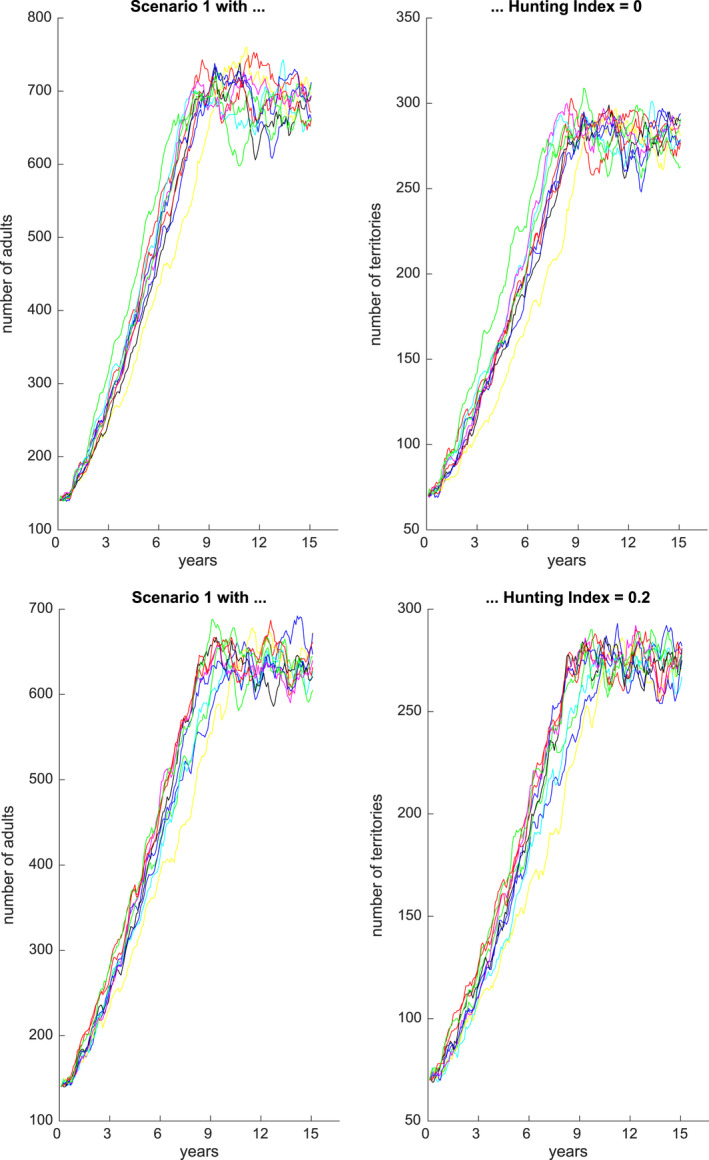
Number of adults (left) and number of occupied territories (right) over 15 years for Scenario 1 and Hunting index = 0 (top) or 0.2 (bottom), based on 10 simulations

**Figure 2:**
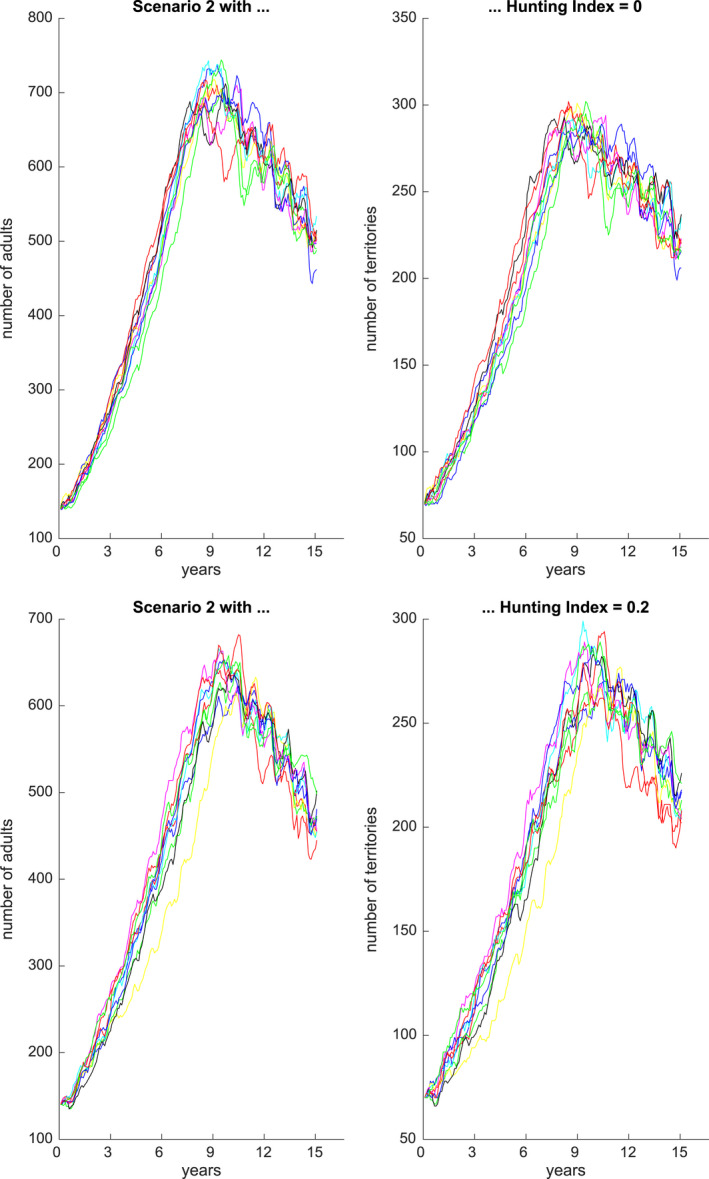
Number of adults (left) and number of occupied territories (right) over 15 years for Scenario 2 and Hunting index = 0 (top) or 0.2 (bottom), based on 10 simulations

**Figure 3:**
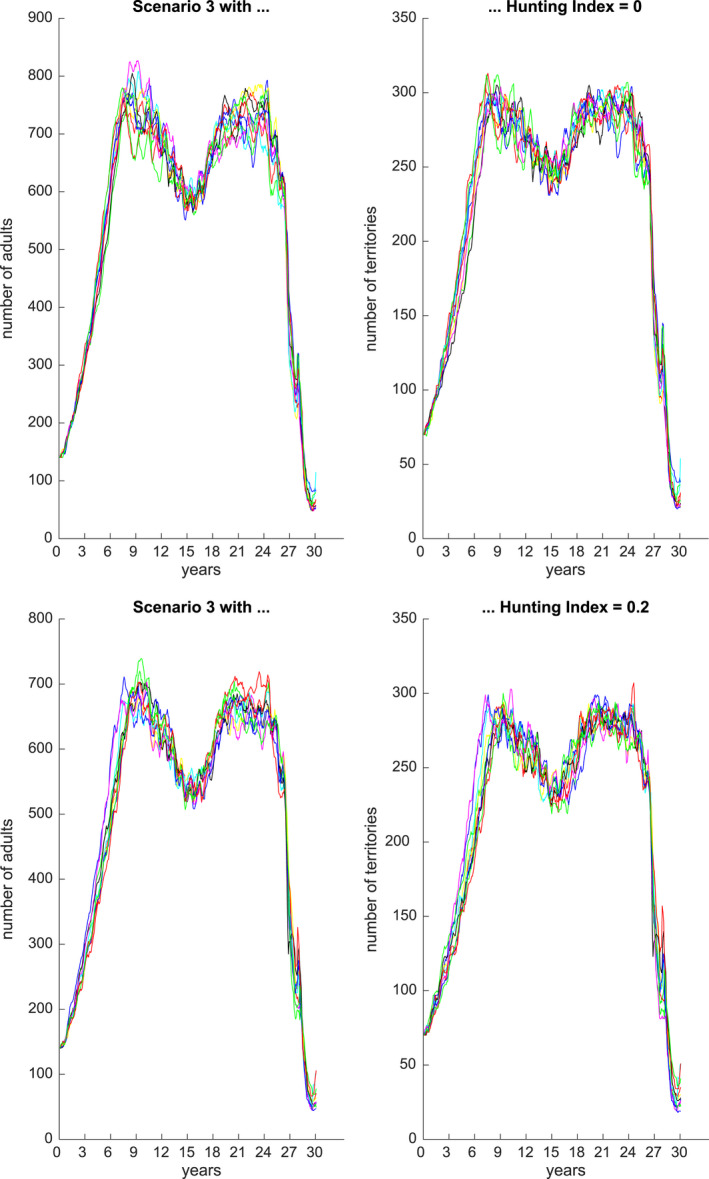
Number of adults (left) and number of occupied territories (right) over 15 years for Scenario 3 and Hunting index = 0 (top) or 0.2 (bottom), based on 10 simulations

**Figure 4:**
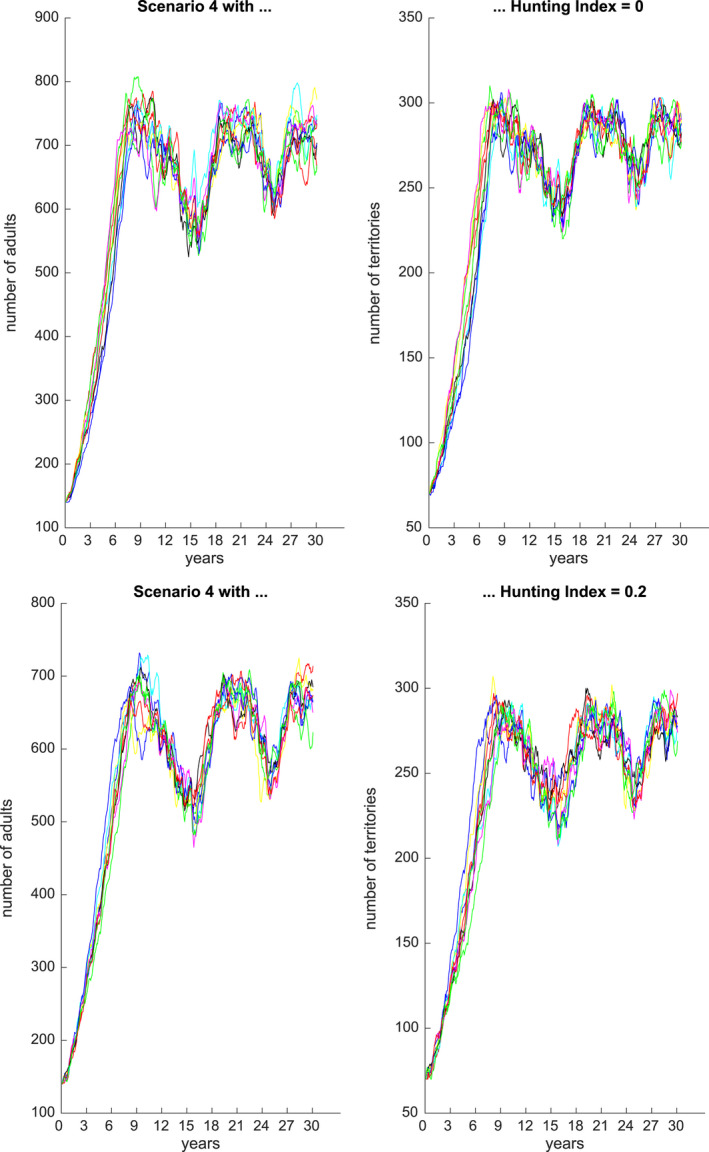
Number of adults (left) and number of occupied territories (right) over 15 years for Scenario 4 and Hunting index = 0 (top) or 0.2 (bottom), based on 10 simulations

**Figure 5:**
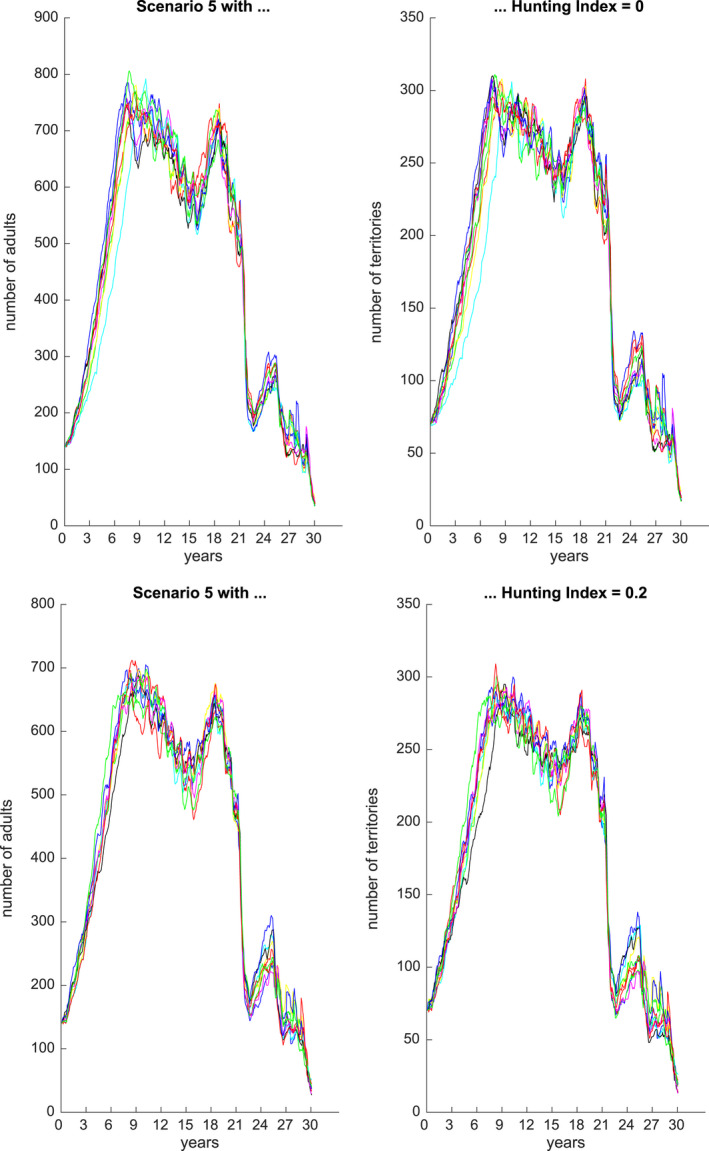
Number of adults (left) and number of occupied territories (right) over 15 years for Scenario 5 and Hunting index = 0 (top) or 0.2 (bottom), based on 10 simulations

**Figure 6:**
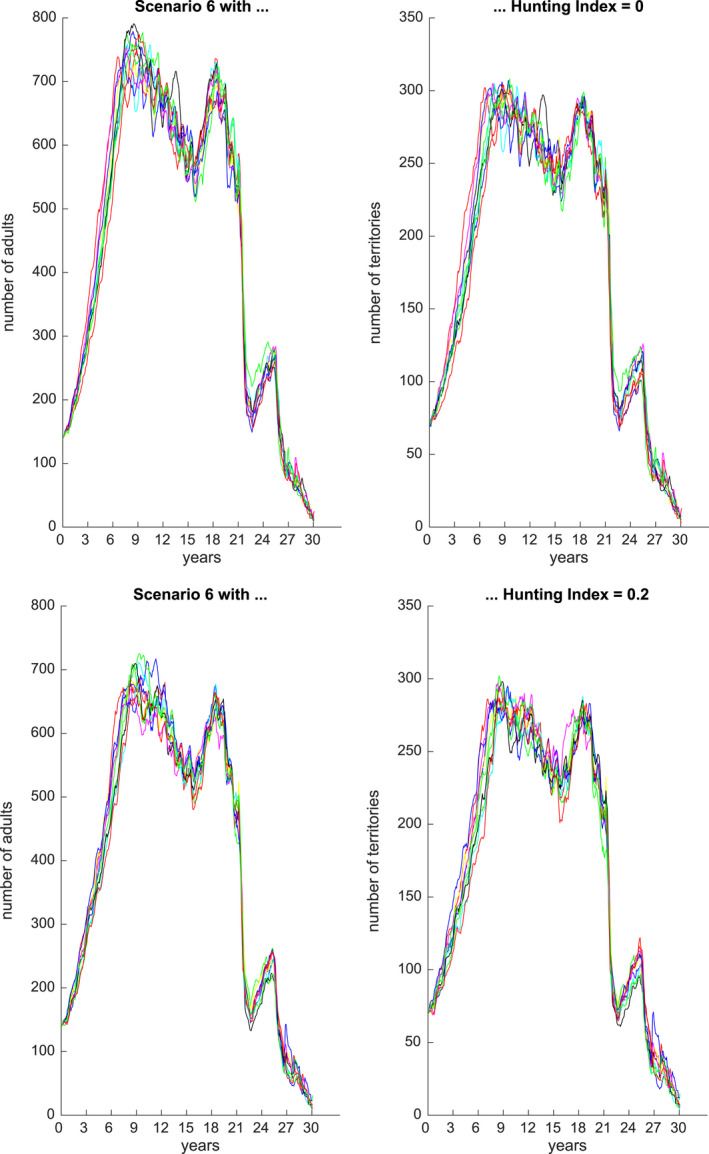
Number of adults (left) and number of occupied territories (right) over 15 years for Scenario 6 and Hunting index = 0 (top) or 0.2 (bottom), based on 10 simulations

Figures 1, 2, 3, 4, 5, 6 each show numbers of adults and numbers of territories over ten different simulations with a hunting index of 0 or 0.2, for Scenarios 1 through 6.

Figure 1 depicts the results of an initial calibration of the model with all scale factors set to 1. Adult population numbers reach their carrying capacity of approximately 700 in approximately 280 occupied territories. With a hunting index of 0.2, the populations are still stable but at a limit of about 50 adults less.

Figure 2 shows adult numbers and territories from 2000–2014 under Scenario 2 based on scaling factors calibrated for 2009–2014 in which there were an extreme drought and three extreme flood events from 2009 to 2014. In the last six years, the number of adults is reduced by approximately 200, and this is also accentuated by a hunting index of 0.2 as for Scenario 1.

Figure 3 shows predicted adult numbers and territories under Scenario 3 over 30 years. We see that when the aquatic animals are not affected then reductions in number of adults (and territories) are less affected, and over five normal years, the numbers can rebound. However, under both extreme flood and drought events, with no recovery periods, then over six years the numbers reduce dramatically to approximately 50. Figure 4 shows predicted numbers under Scenario 4, for a 30 year period. In this setting, there is a sequence of droughts over 6 years then a flood and then two successive droughts. In this case, the population numbers are relatively stable but undergo oscillations. The hunting index has an effect of reducing numbers by approximately 50.

In the case of Scenario 5 (Figure 5), there is an alternating sequence of drought and flood with sufficient stable behavior in between these extreme events so that prey numbers may partially recover, but never fully. Thus, adult numbers attempt sporadic comebacks but the general trend is downwards. As before, the hunting index has an effect on a healthy population but much less so when population levels are low, due to relative effects.

Finally, Scenario 6 (Figure 6) is similar to Scenario 5, but in the final 5 years there are successive years of drought and flood with no recovery, and in this case, the numbers are driven down to single digits or low to mid‐teens.

**Figure 7:**
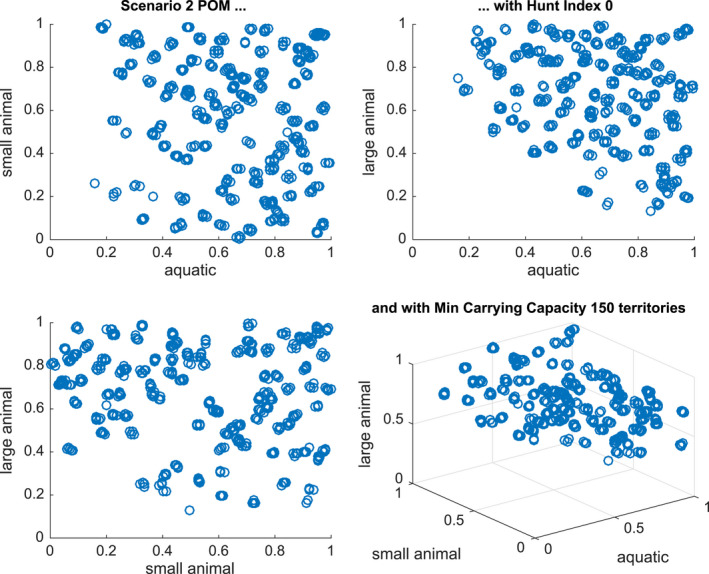
Distribution of parameters in a calibrated population of models for Scenario 2 with hunting index = 0 and with a minimum sustainability of 50\% of the maximum carrying capacity (150 territories) From left to right and top to bottom the Figures show $K_{s}K_{A},K_{L}K_{A},K_{L}K_{S}$ and all three scale parameters

**Figure 8:**
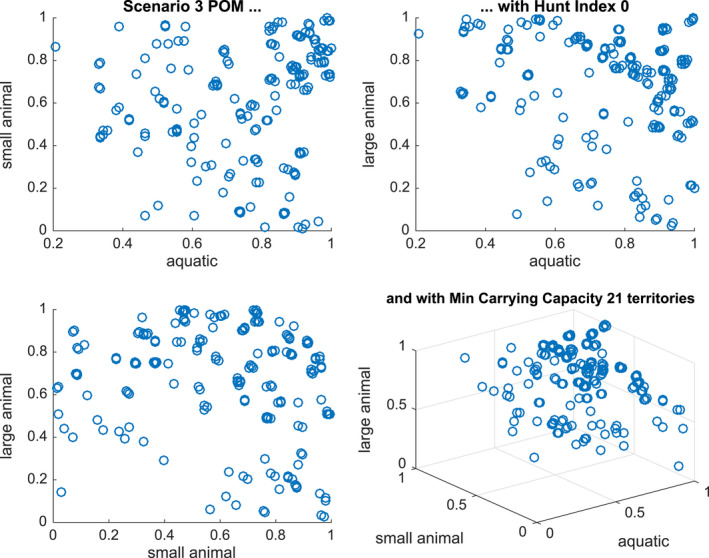
Distribution of parameters in a calibrated population of models for Scenario 3 with hunting index = 0 and with a minimum sustainability of 7\% of the maximum carrying capacity (21 territories) From left to right and top to bottom, the Figures show $K_{s}K_{A},K_{L}K_{A},K_{L}K_{S}$ and all three scale parameters

**Figure 9:**
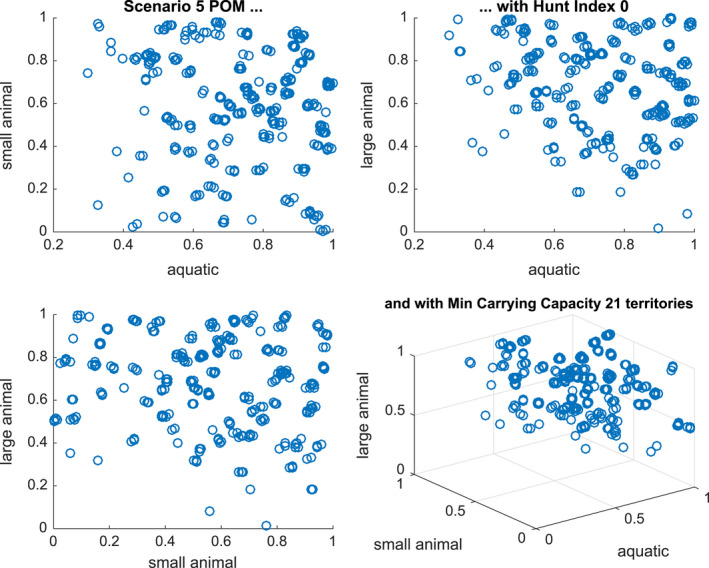
Distribution of parameters in a calibrated population of models for Scenario 5 with hunting index = 0 and with a minimum sustainability of 7\% of the maximum carrying capacity (21 territories) From left to right and top to bottom, the Figures show $K_{s}K_{A},K_{L}K_{A},K_{L}K_{S}$ and all three scale parameters

The results of the population of models study are shown for Scenarios 2, 3, and 5 in Figures 7, 8 and 9, respectively. Since we are building a population over three parameters $K_{A}$, $K_{\mathrm{SL}}$, and $K_{\mathrm{LL}}$ (with H fixed at 0 or 0.2), we present two‐dimensional plots of successful models against each of the three, two‐dimensional cross‐sections, as well as the three‐dimensional plot.

In Figure 7, we present the successful models that generate 50\% of the minimum carrying capacity under Scenario 2. There are 479 models out of the 1,000 tested. We see that there is a pronounced hyperplane below which there are no models. This is very sharp when examining the relationship between the aquatic and large animal indices and suggests that large and aquatic animal numbers are key in wet and dry seasons. This relationship is less obvious in the case of small and large animals.

In Figure 8, we present the 183 successful models out of 1,000 tested that generate 7\% of the minimum carrying capacity under Scenario 3. We see similar relationships as in Figure 8 but now availability of all three classes of animals is important. There is also more of a clustering when all three indicators are high (near 1).

In Figure 9, we present the 297 successful models that generate 7\% of the minimum carrying capacity under Scenario 5. There is again a similar hyperplane structure but in this case if the aquatic animal numbers are very low then there are no successful models. This is consistent with the scenario in which there are alternating sequence of floods and droughts with little recovery in prey numbers.

**Figure 10:**
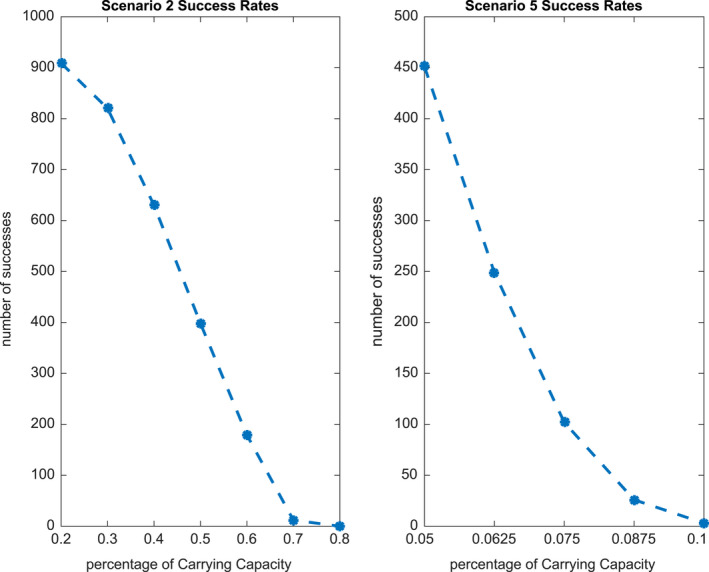
Proportion of successful models achieving the nominated percentage carrying capacity: scenario 2 (left), scenario 5 (right)

We note that for Scenarios 3 and 5, we build a population of models against a very low level of the minimum carrying capacity (7\%). If we were to use higher values, we would see increasingly low numbers of models accepted. In order to understand this effect, we plot the proportion of successful models as a function of the values of the sustainable carrying capacity (Scenario 2, left, and Scenario 5, right) in Figure 10. Examining this figure, we see that the two scenarios are very different. In the case of Scenario 2, there are still successful models with a percentage carrying capacity of 0.7, but for Scenario 5 this percentage carrying capacity value is only 0.1.

### 3.2 Sensitivity assessment

The sensitivity assessment revealed the following insights.

Under Experiment (Sanderson et al., 2002), the change in number of territories resulted in proportional changes in the outcomes of interest (number of animals, number of regions) under both scenarios. Thus under Scenario 1, compared with the obtained maximum values of (700, 280) for 300 territories after 8–9 years, halving the number of territories resulted in maximum values of (350, 140) after approximately 4 years, with much more variation between simulations, and doubling the number of territories resulted in maximum values of (1,300, 530) after approximately 12 years. Similarly, under Scenario 2, the peak values of (680, 280) dropping to (500, 230) under Scenario 2 became (340, 140) dropping to (320, 130) if the number of territories was halved and (1,000, 420) dropping to (620, 280) if the number of territories was doubled.

For the other parameters, under Scenario 1, there was no substantive change in the two outcomes of interest under any of the experimental setups (i.e., under any of the changes in underlying parameters).

For Scenario 2, compared with the obtained values (nominal maximum values of (600, 280) dropping to (500, 230), minor changes were observed as follows:

Shorter lifespan: slightly more dramatic effect (maximum values of (600, 280) dropping to (500, 230) under lifespan of 15 years; (650, 280) dropping to (450, 210) under lifespan of 10 years). Change in birthrate: slightly larger numbers of animals (maximum 700) and territories (300), dropping to 600 and 280, respectively. Change in hunger threshold: slightly larger maximum number of regions (700) but no change in other outcomes. Change in food requirement: same maximum values, less dramatic drop (to 600, 260) when food requirement was halved and more dramatic drop (to 320, 150) when food requirement was doubled.

**Figure 11:**
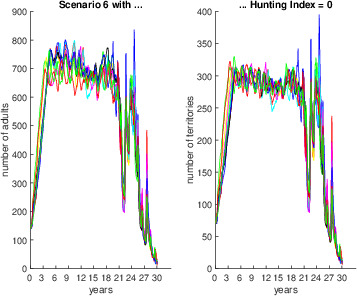
Sensitivity analysis for Scenario 6, assuming a breeding season twice as long as in the main simulation (6 months instead of 3 months)

For Scenario 6, although there were some minor variations, the same overall patterns were observed under all sensitivity experiments, and all populations crashed. For example, under Experiment (Caso et al., 2011), a longer breeding season resulted in greater oscillation between simulations, especially after a simulated period of 20 years, with the population crashing to zero by 30 years (see Figure 11).

## 4 DISCUSSION

In this paper, we have developed a stochastic temporal modeling framework that allows us to assess patterns of change in the number of predators, here jaguars, in response to food availability and possible future climate scenarios. The underpinning model is a comprehensive discrete Markov model of jaguar populations in the case study location of Pacaya Samiria. We modeled six jaguar population scenarios taking into account solitary behavior, mating, births of cubs at certain times of the year, competition, illegal hunting by humans, and death from starvation, including availability of key prey.

Scenario 1 estimated the jaguar population size in the Pacaya Samiria reserve in Peru, assuming stable prey availability. The jaguar population was estimated to stabilize around 600–700 adult jaguars, in 250–300 occupied territories. This estimate gives a population density of approximately 2.88–3.37 jaguars per 100 km^2^, over the total 20,800km^2^ size of the reserve. Our model thus provides jaguar density estimates slightly lower than, but still comparable to, previous approaches using camera trap analysis in the nearby long‐term monitoring areas of Tambopata and Espinoza in the Peruvian Amazon (4.4 per 100 km^2^) (Tobler et al., 2015), as well as other estimates from camera trap population studies of jaguars in open lowlands in Brazil (2.67 per 100 km^2^) (Silveira et al., 2010; Silver et al., 2004) and Venezuela (4.44 per 100 km^2^) (Jedrzejewski et al., 2017). The presented computational simulation method could therefore provide an alternative mechanism to evaluate jaguar population distributions in areas where abundance data, such as camera trap surveys, are not available.

Scenario 2 estimated the jaguar population size in the Pacaya Samiria following changes in prey populations resulting from extreme weather conditions experienced in the western Amazon from 2009 – 2014 in which there was one extreme drought and three extreme flooding events. Scenario 2 estimated a 6‐year population size similar to Scenario 1, but following this series of extreme weather events, the jaguar population was estimated to decline and stabilize around 500 jaguars in the Pacaya Samiria, or a population density of approximately 2.40 jaguars per 100 km^2^. This estimate is closer to that calculated for jaguars in the adjacent Madidi National Park in the Amazon rainforest region of Bolivia (2.8 per 100 km^2^) (Silver et al., 2004), where jaguars faced a relatively low abundance of prey species due to hunting (Wallace, Gomez, Ayala, & Espinoza, 2003). Our model therefore estimates that jaguar populations could have declined by up to 30%, due to extreme weather events impacting prey availability in the period 2009–2014.

Scenarios 3–5 explored alternative futures for jaguar populations in the Pacaya Samiria under different climate scenarios, in which prey populations partially recover between different sequences of floods and droughts. In Scenario 3, jaguar numbers recover to almost 800 jaguars over five normal years, but after a subsequent series of alternating droughts and floods, the population declines rapidly to only 50 jaguars in 30 years. In Scenario 4, where the extreme weather events are mostly floods and aquatic prey remains available, the jaguar population remains stable but oscillates between 500 and 800 jaguars over the 30‐year period. In Scenario 5, with alternating drought and flood events in which prey levels recover partially but never fully, jaguar populations show a series of declines and partial recoveries, but leading to an overall decline from 700 to less than 50 jaguars in 30 years.

Scenario 6 was an extreme case in which over the last 5 years drought and flood occurred every other year and prey levels could not recover. In this case, jaguar populations showed a sharp decline after initial extreme weather events, and with subsequent droughts and floods with no prey recovery, the jaguar population was predicted to drop to zero by year 30.

The scenarios additionally explored the effect on jaguar populations of humans hunting jaguars. In all scenarios, the effect of human hunting was low compared to extreme weather events and prey availability. When jaguar populations were healthy, human hunting had an effect on the overall population size, but not on population stability. When extreme weather events drove jaguar populations to low levels, there were fewer jaguars to hunt, and therefore hunting levels were relatively low. However, the model did not analyze the additional pressure on jaguar populations from human hunting of prey species. Given the importance of prey availability to jaguar population stability following extreme weather events, hunting of prey species may impact jaguar populations as suggested in (Wallace et al., 2003).

In addition to constructing a stochastic model of jaguar abundance, this paper has demonstrated a generic framework for modeling problems that have significant uncertainties in the underlying processes as well as coping with highly variable and sparse data. The population of models study presented here allowed us to evaluate the robustness of the derived results to inherent uncertainty and variability within each scenario. We used a stochastic modeling approach that considered a range of simulated models under a distribution of starting parameters. This approach accounts for the intrinsic uncertainty of baseline numbers on jaguar population density, territory size, and prey availability. We additionally tested the sensitivity of our model results to variations in the baseline model assumptions and found that, overall, our models were robust to the starting assumptions and base data.

We note, however, that some of our conclusions are still predicated on assumptions inherent in the models. For example, we assume that the scale factors for small and large animals are the same. If they were not equal then we should see more discriminatory effects between small and large animals. However, this would come at the cost of having to quantify even more uncertainty in the models. Furthermore, there is little information in our data to suggest that these particular scale factors should be different.

Another assumption is that the case study region is closed, in that there is no emigration or immigration of jaguars or prey from other regions. Although this is unlikely, if climate variation affects contiguous regions similarly then such an assumption may be reasonable—for example, low prey in this region would be matched with low prey in neighboring regions and movement into the region would be matched with movement out of the region, assuming initial stationarity and all other things being equal—and hence, the results found here may still be feasible.

Our results are concerning for the future viability of jaguar populations in the Peruvian Amazon under climate change scenarios. Our model predictions support previous findings that the Peruvian Amazon is, currently, a core habitat for jaguars (Tobler et al., 2015; Wallace et al., 2003). Predictions show that jaguar populations can recover after extreme weather events, provided that there is enough time between extreme weather events for prey populations to stabilize. However, a series of rapid extreme weather events causes jaguar populations to oscillate, and if the prey population does not have time to recover between weather events, these oscillations can lead to rapid jaguar population decline. Under climate change, the frequency of extreme weather events is expected to increase (Bodmer et al., 2015; Cook et al., 2012). According to our simulations, an increase in the frequency of extreme weather events due to climate change would cause rapid and irrevocable decline in the jaguar population of the Peruvian Amazon.

Overall, our results imply that jaguar populations exhibit some robustness to extreme drought and flood but that they can decline to low levels if subject to a succession of these events over short time periods. These declines may be further exacerbated by hunting. Our model also suggests that jaguar numbers can return to stable populations if there are periods in which climate patterns are more benign and other factors are conducive, albeit possibly at much lower numbers.

## CONFLICT OF INTEREST

The authors have no competing interests to declare.

## AUTHOR CONTRIBUTION

**Kevin Burrage:** Conceptualization (lead); Data curation (lead); Formal analysis (lead); Methodology (equal); Writing‐original draft (lead). **Pamela Burrage:** Formal analysis (equal); Investigation (equal); Writing‐review & editing (equal). **Jacqueline Davis:** Investigation (equal); Writing‐review & editing (equal). **Tomasz Bednarz:** Investigation (equal); Writing‐review & editing (supporting). **June Kim:** Investigation (equal); Writing‐review & editing (supporting). **Julie Vercelloni:** Investigation (equal); Writing‐review & editing (supporting). **Erin Peterson:** Investigation (equal); Writing‐review & editing (supporting). **Kerrie Mengersen:** Conceptualization (equal); Formal analysis (supporting); Funding acquisition (lead); Project administration (lead); Writing‐review & editing (lead).

## Data Availability

Data sharing is not applicable to this article as no new data were created or analyzed in this study. Material on the project can be accessed at http://vis.stats.technology
